# Methods of Current Practice: Qualitative Analysis of Intervention Strategies Utilized by Vaccine Waiver Educators in Michigan

**DOI:** 10.1177/15271544221114293

**Published:** 2022-08-10

**Authors:** Duduzile P. Mashinini, Mary D. Lagerwey, Kieran J. Fogarty, Rachel C. Potter

**Affiliations:** 1 College of Health and Human Services, Interdisciplinary Health Sciences Ph.D. Program, 4175 Western Michigan University, Kalamazoo, MI, USA; 2 College of Health and Human Services, Bronson School of Nursing, 4175 Western Michigan University, Kalamazoo, MI, USA; 3 4410 Michigan Department of Health and Human Service (MDHHS), Division of Immunizations, Lansing, MI, USA

**Keywords:** vaccine, education, exemptions, vaccine communication, mandates, vaccine policy

## Abstract

By enacting administrative rule 325.176 (12), Michigan added a vaccine education component as a precondition to granting vaccine waivers to vaccine-hesitant parents wishing to file a nonmedical vaccine exemption for their school-aged child. The purpose of the study was to identify best practices for reaching vaccine-hesitant parents during face-to-face vaccine education sessions conducted by vaccine waiver educators in Michigan. This study utilized qualitative descriptive content analysis of semi-structured phone interviews with vaccine waiver educators from local health departments (LHDs) in Michigan. Participants were vaccine waiver educators who were employed by a local health department in Michigan and had conducted at least 30 vaccine waiver education sessions. Strategies, resources, and techniques identified by educators as beneficial included using and providing information from a variety of sources, compiling their own educational materials, creating a positive experience, holding personalized sessions, and streamlining exemption and vaccination sessions. However, unexpected themes that emerged from the interviews revealed that vaccine waiver educators need additional training in discussing vaccine ingredients with parents, handling religious vaccine exemption requests, and assessing the role of schools. Implementing successful vaccine education interventions targeting vaccine-hesitancy is crucial to public health. Charging LHDs with overseeing vaccine education via a face-to-face discussion is a novel intervention strategy, the effective implementation of which may inform vaccine education intervention nationwide and may even be translated into international contexts and prove useful to current COVID-19 vaccination efforts.

## Introduction

Vaccine education has been an extensively utilized strategy for helping to inform vaccine decision-making (Kaufman et al., 2018; Li et al., 2021). Through administrative rule 325.176 (12), Michigan health officials adopted the vaccine education strategy by adding a face-to-face educational component at local health departments (LHDs) for parents seeking nonmedical vaccine exemptions (waivers) for their school-aged children (Michigan Department of Health and Human Services [MDHHS], 2015, 2017). With this measure, Michigan joined several other states in introducing an educational component as a precondition for attaining a nonmedical vaccine exemption for school entry (Centers for Disease Control and Prevention [CDC], 2015). However, of the states with policies stipulating an educational component, Michigan is among a few to have assigned the responsibility to LHDs (CDC, 2015). Michigan is the only state implementing the health department-based vaccine education program via a face-to-face discussion with a vaccine waiver educator (Navin et al., 2018). Being that they already oversee community health needs and staff direct care nurses, Michigan LHDs seem well equipped for this task.

Several studies have been conducted assessing the impact of Michigan's administrative rule on vaccine exemption rates (Mashinini et al., 2020; Masters et al., 2021; Navin et al., 2020), but very few have directly focused on the implementation of the vaccine education sessions. One of the earliest studies focusing on the implementation of the vaccine education session in Michigan was conducted by Largent et al. (2015). A key finding from Largent et al.'s pilot study was the vaccine waiver educators' belief that roughly 30% of parents that educators spoke to were willing to reconsider their vaccination decisions because of the sessions. Vaccine waiver educators in Largent et al.'s study also stated that about 10.5% of parents verbalized to them that they wanted their child to receive the vaccine they previously wanted to waive. Part of the aim of Largent et al.'s study included understanding how vaccine waiver educators were implementing the education sessions, and to begin examining best practices for conducting the sessions. The results of these goals, however, were not fully conveyed in the online presentation slides. One study that addressed this was conducted by Navin, Kozak, & Clark, in 2018, who revealed notable themes that speak to best practices in their qualitative study assessing educators’ experiences conducting the education sessions. The vaccine educators in Navin et al.'s study realized the difficulty of changing parents' minds, so their goals evolved from seeing the sessions as a mechanism to change parents’ minds to one focused on “planting a seed” through relaying accurate scientific information. Other themes related to best practices found in this study included adopting a conflict reduction and customized approach for each unique parental interaction, as well as strategies for addressing the emotional toll the sessions had on educators. Navin et al.'s 2018 study contributed substantially to understanding best practices for vaccine waiver education; however, this was not the study's primary goal, and more work is needed in this realm. Furthermore, as the program is ongoing, it is important to continue to pursue studies that illuminate what is working for vaccine waiver educators during these sessions, and what can be improved. Consequently, the current study can be viewed as an extension of both Largent et al., and Navin et al.'s 2018 studies, but one that focuses specifically on highlighting resources, strategies, and techniques used by vaccine waiver educators when conducting vaccine education sessions.

The purpose of this study was to identify and describe vaccine waiver educators’ views on what they found to be best practices in their education of vaccine-hesitant parents. An analysis of educators’ experiences with planning for and providing these face-to-face education sessions could not only enhance the viability of the program but also be utilized as a guide for public health professionals who are looking for nuanced approaches to engaging vaccine-hesitant parents.

### The Current Vaccine Exemption Process

Although it varies by health department, the vaccine waiver process at local health departments in Michigan now entails parents contacting their LHD to schedule their educational session. To adequately address objections and concerns, parents may be asked during their initial contact with the LHD to indicate which vaccines they wish to exempt their child from and why (W. Byers-Phillips, personal communication, 2015). Based on the initial information gathered, the vaccine waiver educator conducting the educational session assembles talking points to use during the face-to-face session (Largent et al., 2015). At the end of the session, parents who wish to pursue the vaccine exemption are provided with the official state-certified nonmedical waiver form (DCH-0716), which they deliver to the child's school (MDHHS, 2015, 2017).

### Vaccine Waiver Education Training

Though the state of Michigan did not provide specific implementation strategies for the vaccine waiver education sessions, the state did train educators via webinars, phone, and biannual Immunization Action Plan meetings. Currently, the state provides online resources found on the MDHHS website. Resources include recommendations for tailoring education sessions and addressing specific parental concerns and questions regarding vaccines (W. Byers-Phillips, personal communication, February 8, 2015; Author, personal communication, October 22, 2018). Resource guides provided by the state cover myriad vaccine-related topics for educators preparing the educational sessions for parents with philosophical objections (MDHHS, 2017). Among other things, MDHHS online resources include communication strategies for dialoguing with vaccine-hesitant parents and Vaccine Information Statement (VIS) fact sheets furnished by the CDC. Online resources also provide educators with videos highlighting “good conversation points,” and some showcasing vaccine preventable diseases [VPD] stories, which the website claims are “a communication strategy shown to be effective when communicating with vaccine-hesitant parents” (MDHHS, 2017). Initially, the state also included talking points pertinent to education sessions for those seeking religious exemptions; however, due to judicial challenges to the state's ability to challenge parents’ religious beliefs protected by the free exercise and establishment clause, the state decided halfway through the second year of the policy to no longer engage in discussions with parents seeking a religious exemption. This change, while eliminating the discussion, did not change parents’ ability to file a religious exemption, thus making it easier for parents to file a religious exemption as opposed to a philosophical one.

Even though the training is still conducted upon request and issues regarding the sessions are brought up in biannual and monthly local health department meetings, the extent to which vaccine waiver educators utilize resources and employ strategies and techniques to reach vaccine-hesitant parents has not been fully researched. Therefore, this study asked what resources, strategies, and techniques do vaccine waiver educators use and find most effective in reaching vaccine-hesitant parents during educational vaccine sessions?

## Methods

### Study Design

The study used a qualitative research design that employed descriptive content analysis of semi-structured personal phone interviews with vaccine waiver educators. Using qualitative description that “stay[s] close to the data” (Sandelowski, 2000, p. 334) allows for an authentic description of events by relaying the everyday language participants used to describe their experiences with educating parents about vaccines (Bengtsson, 2016; Sandelowski, 2000, 2010). The study was approved by Western Michigan University's Human Subjects Review Board (HSIRB) and by the Michigan Department of Health and Human Services Institutional Review Board (IRB).

The interview questions for this study were informed by the literature review and by a review of existing surveys, including one created by Largent et al., for their 2015 study conducted at two local health departments in Michigan following the administrative rule change. Interview questions for this study were also informed by those used by California's Department of Public Health (CDPH) to assess communication approaches and needs of immunization providers (California Department of Public Health [CDPH], 2012). Interview questions focused on what vaccine waiver educators prefer and use. The specific content of interview questions is included with the findings.

### Sampling Procedure

A purposive sampling strategy was employed in which participant recruitment sites were chosen based on Arc GIS optimized hotspot analysis using ArcGIS version® 10.2., revealing areas where schools had high and low nonmedical vaccine exemption rates (Palinkas et al., 2015). To analyze recruitment sites, we used MDHHS Kindergarten Immunization Status data from report years 2016 and 2017 acquired through a data use agreement with MDHHS.

Immunization Action Plan (IAP) coordinators from optimized-analysis-selected sites across the state were made aware of the study by MDHHS via email and were informed that their health department may be invited to participate in the study. To minimize bias, hot-spot-analysis results for participant recruitment were not shared with the recruited health departments and are not revealed for anonymity.

Supervisors at local health department sites identified by the Arc GIS hot-spot-analysis tool were contacted and asked if their department would like to take part in the study, and if so, for permission for the primary researcher, a Ph.D. student from Western Michigan University's Interdisciplinary Health Science program studying vaccine exemptions, to recruit educators at their sites to participate in the study. The final sample size was governed by availability and data saturation (Morse, 2015; Palinkas et al., 2015). Interviews were conducted in July and August of 2018. Participation was voluntary, and participants’ responses were kept confidential to encourage them to speak candidly without concern for possible repercussions.

The primary investigator contacted only those participants who signed the written informed consent form via electronic signature to schedule a date and time for an interview. Interviews were conducted by the primary researcher via phone and recorded using a digital recording device, as well as a second backup recorder. Interviews were conducted during working hours; therefore, participants were interviewed while at their place of employment. The interviews took 30–50 min. Participant responses to the interview questions were kept confidential, and pseudonyms were used during analysis.

### Participants

The study included seven female participants from four different local health departments. All participants were nurses, except for one who held the title of a health educator. Two participants had been at their current positions for less than three years, with the remaining five reporting over 10 years of service at their current position. All participants reported having routinely conducted vaccine education sessions since at least 2015; the least experienced participant reported having conducted at least 30 sessions.

### Qualitative Analysis

We applied a manifest analytic approach to the descriptive content analysis to identify current methods of vaccine waiver education from the perspective of individual educators (Bengtsson, 2016). All recorded interviews were stored on an encrypted flash drive and were transcribed verbatim by the primary researcher. The backup recordings were also uploaded to the encrypted flash drive and then erased from the backup recording device after they were transcribed and checked for accuracy. Upon transcription of the interviews, pseudonyms were used to replace participants’ names and any names they divulged during the interview. All results were reported in a manner ensuring that data would not be traced back to an individual.

Analysis of interviews included note taking and a thorough re-reading of all the transcribed interviews before the coding process. The coding process was informed by the data via complete data immersion. Coding was informed by the research question encompassing two categories, resources, and strategies/ techniques. The primary researcher conducted initial coding. For inter-rater reliability, samples of the transcribed interviews and codes, tracked in a codebook, were reviewed by co-authors and were compared for agreement.

Initial coding focused on characteristics of vaccine waiver educators’ personal strategies/ techniques, and resources used for navigating Michigan's administrative rule change for face-to-face encounters with parents. An additional code of local health department-based vaccine educational needs emerged.

## Results

Two themes emerged related to resources, and three themes emerged pertaining to strategies and techniques (Table 1). The first theme related to resources included using and providing a variety of resources, and the second related to resources was compiling their own educational materials (both as a guide for conducting sessions and as a handout for parents). Themes falling under the strategies and techniques category were creating a positive experience, conducting personalized sessions, and streamlining exemption and vaccination sessions. Three additional themes emerged from the interviews which were not part of the research question (Table 2): educators voiced a need for further support, in the form of training, regarding vaccine ingredients, religious exemptions, and the role of schools.

**Table 1.** Themes and Quotes.

Categories	Themes	Participant Quotes
Resources	Using and providing a variety of resources	“I make sure that when I share resources with them, it's from a variety of different resources.” “If the person's anti-CDC or government we just give them info from the Children's Hospital of Philadelphia (CHOP), and from Immunization Action Coalition (IAC).” “The.orgs or the edu., is usually what we try to reference instead of the governmental.” Yeah, I kind of feel out their education level and how much information they’re seeking. I don't just throw a bunch of papers at them. I don't want to overwhelm them.” “We use a lot of Children's Hospital of Philadelphia. They have a lot of information. They have a Q&A section about childhood vaccines and of course, it all depends on the educational level too. Some things are more simple than others. We use Immunization Action Coalition. They have a lot of good handouts. We use the CDC's [pamphlet] ‘If You Choose not to Vaccinate Your Child, Understand the Risks and Responsibilities’”
	Compiling their own educational materials	“We didn't use information directly from the state, but we’ve used their guidance and developed our own.” “We’ve developed our own double-sided handout about why you should vaccinate your child” “We have also developed our own messages too like a little tip sheet; we have combined a lot of this information. We have our own CD that talks about the incubation period of the diseases and how long they are communicable.”
Strategies & Techniques	Creating a positive experience	“To me [a successful session] is not necessarily based on if they change their mind. To me, it's if we have had a good open dialogue and the parent felt that they could express their concerns…it's an open dialogue where neither party is frustrated or felt they weren't heard.” “You start the conversation in whatever form of words, information, posture, or interaction that communicates that we know that the parent wants what's best for their kid…we just try to treat parents with respect and acknowledge that they are a good parent, and usually that is a big help.” “I keep it a very friendly atmosphere, and my reason is that I really want them to come back…and I’ve had people come back.”
	Personalized sessions	“It is very personal, and we keep it personal [so] that we have a dialogue that will be meaningful.” “I try to have at least some sort of personal story, and if it's not about me, it's about someone I know, because I think that helps.” “I use it [a personal story] in regard to hepatitis B in relation to a sports-related injury. I talk about how my children were always the first ones to help and they were always the ones with the blood on their hands because they were just those kinds of kids, but some parents just don't think about that.” “We talk about how VPDs are on the rise, we talk about local statistics, local outbreaks that we’ve had here, in a way to drive home the point that these [diseases] are still out there and if your children are not protected, then they’re most at risk.”
	Streamlined Exemption and Vaccination Sessions	“We make appointment times the same for a waiver appointment as we do for an immunization appointment, so it [the rule change] hasn't added to the workload at all. It's part of our normal workday.” “Making the process [for obtaining a waiver] the same [as the process for obtaining a vaccine] is the most beneficial.” “Our appointments are 25 min for everything. So, they [exemptors] get the same appointment time slot that someone coming in for immunization would get.”

**Table 2.** Unexpected Themes and Quotes.

Unexpected Themes	Participant quotes
More training regarding vaccine ingredients	“…when they throw out a strange off-the-wall ingredient and they know that this chemical is a bad thing, you know…I’m not the scientist behind the vaccine manufacturing so I might not be able to answer that question specifically.” “…they go on and on about the neurotoxins, the metals, and the heavy metals and how they’re metabolized…a couple of those get really fine-tuned with their questions and I’m not a pharmaceutical person.” “The hardest one is when people argue [about] the ingredients in the vaccines because it is what it is. I can't change the ingredients.”
Religious exemptions	“I think there's just a really small percentage [of religious exemptions] that are truly religious,” “I think some [parents’ requests for waivers] come in under the umbrella of religion because they know that I’m not going to ask any more questions.” “Most of my people aren't refusing all vaccines the ones that are know to put ‘religious’; they used to put ‘philosophical’, but now they know to put ‘religious’ because once they put ‘religious’, we kind of stop there.” “…sometimes they will write down ‘religious’ even though we’ve discussed it and it's not really religious. People will put down that they don't believe in vaccines and they see that as a religious reason.”
The role of schools	“Some schools will say you have about a week [to present documentation on vaccinations]. Now the kid has been in school for a week and then they are out, so now what? What have you really accomplished but having people really ticked off?” “…there are counties that don't practice what the code says, which makes it really tough.” “I do feel like some schools give them extra time. [In]consistent messaging, I think, is a constant issue.”

### Themes

#### Resources

##### Using and Providing a Variety of Resources

The theme of using and providing a variety of resources was found in interviews with all but one participant. Among some participants, using a variety of resources was described as part of a strategy for establishing credibility with parents. When asked which materials/resources provided by MDHHS, did she use or reference most often, and which she used outside of those provided by the state, a participant explained that when selecting resources to share with parents, she purposefully sought out resources furnished by different organizations so, *“they’re not coming from the same direction. You need to have multiple resources that say the same things in different ways.”*

Participants also talked about the necessity of drawing from a variety of resources to navigate mistrust of government expressed by some parents, with some finding themselves explicitly refraining from using or providing governmental resources such as those furnished by the CDC. Using a variety of resources was also a strategy to make certain that the message was clearly received by all parents, regardless of their level of health literacy.

The top three most cited resources used by educators were those from the Immunization Action Coalition (IAC), Children's Hospital of Philadelphia (CHOP), and the Centers for Disease Control and Prevention (CDC). Almost all participants attested to using the CDC's Vaccine Information Statements (VIS) as a handout. Although the distribution of the VIS sheets is not mandated by the state, participants spoke of having a sense that they were “required to” do so.

##### Compiling Their own Educational Materials

When asked about handouts they provide to parents, a majority of educators stated they developed their customized handouts and educational tools both to help guide the sessions and to distribute to parents. Compiling customized education material was also a way to not overwhelm the parents with too much information, with some educators simply providing “*a list of reliable websites”* for parents to reference. Some participants customized their education material to condense the copious amount of information *“all on one sheet.”* Some customized tools addressed the risks and benefits of vaccination, covered local and state statistics, provided information about what to expect if parents choose not to vaccinate, and provided information regarding school exclusion.

#### Strategies and Techniques

##### Creating a Positive Experience

All educators reported that a majority of their encounters with parents were mostly positive, an outcome that, according to several participants, may be driven in part by their strategic approach of having an open dialogue that focuses on building a relationship with parents and emphasizing that the session was *“just going to be a conversation.”* This approach, one interviewee noted, was stressed in a webinar training provided by the state.

When asked what they considered a successful session, many participants believed that having an open dialogue characterized a successful session, in which “*the parent was engaged and asking questions*.” However, some educators reported that this was best facilitated by first acknowledging that the parent is *“a good parent trying to make the best decision”* for his or her child and framing the educational session as *“a tool to help them make that decision.”* Measuring successful sessions in this way may be a proxy for assessing impact and parents’ behavioral outcomes, as participants reported that only a few parents changed their minds during the sessions, and for most, there was no means to follow up on parents’ final decisions regarding vaccination.

Participants reported that some parents responded negatively to the sessions; however, educators were steadfast in their attempts to remain respectful to parents: *“we just try to handle it and not get to their level of emotion…we are polite.”* Although educators noted that only a minority of their encounters with parents were negative, the strategy of creating a positive experience, they believed, not only helped to limit confrontation but also increased the likelihood that the parents would return to the health department.

The notion that educators wanted parents to return speaks to their desire to establish trust and form good long-term relationships between parents and the health department. Some educators expressed frustration with other educators who they believed were not dialoguing in good faith, which they felt undermined their overall mission. Commenting on this, one educator said, *“I think what's happening is that there are nurses that are making people mad and then people are turning around and suing the state and hurting us in the long run.”*

##### Personalized Sessions

When asked about the format in which their health department conducted these sessions, almost all educators expressed a belief that individual sessions were preferable to group sessions; the individual sessions allowed educators the opportunity to personalize the experience as much as possible, believing that *“there is not a one-size-fits-all ever!”* This individualized approach complements the educator's attempt to establish a good relationship that fostered trust and showed respect.

When asked how often they draw from their own personal experience, all participants, except for one, indicated that they tried to do so as often as possible during the sessions *“to make the interactions with parents more personal*.” Some participants cited their use of state and local community level statistics regarding vaccine-preventable disease incidence and outbreaks as part of their strategy for personalizing the sessions, emphasizing how close the issue is to home. A participant reported showing parents the *“Michigan vaccine-preventable disease summary [report] webpage to show the specific VPDs occurring in the local community.”*

#### Streamlined Exemption and Vaccination Sessions

When asked how the sessions affected workload, all but two participants stated that the vaccine education sessions had added little to their workload though they endorsed the practice of blocking off equal time for vaccine waiver education sessions and vaccination appointments to limit disruptions to workplace productivity. This strategy ensured that participants were not making it easier for parents to get an exemption than a vaccination, reinforcing one of the primary objectives of the program (Lillvis et al., 2020). Streamlining the exemption and vaccine sessions was deemed a useful strategy that equalized the burden of exempting and vaccinating: *“We don't want to make it easier for someone to get a waiver.”* A participant who noted that her health department is in a high waiver area and processes at least 45 waivers a day concurred with the benefits of streamlining the vaccine and exemption sessions:*“Our appointments are 25 min for everything. So, they [exemptors] get the same appointment time slot that someone coming in for immunization would get.”*

##### Emerging Themes

Three major unexpected themes emerged during the interviews, outlining areas that participants felt needed refinement and where they required additional training and support (Table 2).

##### More Training Regarding Vaccine Ingredients

Inquiries into whether educators felt they received adequate training revealed that, overall, participants felt they initially received adequate formal training to conduct the education sessions; however, when probed further about what aspects of training went well, several participants disclosed that on-the-job training that enabled them to sit in on a session and observe a colleague was the best way for them to learn how to conduct the educational sessions. In response to the question asking about improving the training, four of the seven participants verbalized feeling inadequately prepared to answer parents’ questions regarding vaccine ingredients. When asked which parental concern they felt least adequately prepared to address, one participant responded by saying, *“when they throw out a strange off-the-wall ingredient and they know that this chemical is a bad thing, you know… I’m not the scientist behind the vaccine manufacturing so I might not be able to answer that question specifically.”*

##### Religious Exemptions

All participants talked about complications regarding religious belief exemptions and the ramifications of not being able to converse with parents filing for religious belief exemptions: “… *once they say, ‘Oh, it's religious,’ we’re not allowed to ask any more questions*.” Some voiced concerns about the increased frequency of parents asking for a religious exemption, as a way of circumventing a discussion with health officials: “*lately, religious [exemption] is gaining ground because there's sort of a grassroots movement […] [P]arents can come in and say, ‘because of my religion’ […] [T]hey know that we’re not going to have much to say.”*

Some participants believed that some parents did not truly understand what a religious exemption signified and incorrectly categorized their exemption request.

##### The Role of Schools

Although all participants talked about their health department providing many training sessions to schools, conducting several meetings throughout the year with all school personnel involved in the vaccine waiver process, and keeping personnel abreast of information through regular emails, many participants expressed a belief that the role of schools in the nonmedical vaccine exemption process gravely needs reform. Participants concurred that getting schools statewide to be more consistent in following state legislation on the first day of school exclusions for children whose parents did not have adequate documentation was essential to the successful enforcement of the policy. Some expressed that schools “*need to be tougher*” regarding first-day exclusion because some schools “*do let them slide a bit*.” An educator noted a need for balance, adding that keeping in close contact with the schools and having “*a good relationship with them*” is helpful.

## Discussion

This study outlined several best practices for conducting vaccine waiver education sessions with vaccine-hesitant parents. The first was using and providing a variety of resources. Keenly recognizing the element of government distrust that often underscores vaccine hesitancy, participants advocated for the use of other scientific-based sources, such as IAC and CHOP, within these resources. Compiling their own materials for conducting the sessions as well as producing their own handouts to distribute to parents supported efforts to expose parents to a variety of sources. Unexpectedly, given their comments about having adequate education on vaccines, several participants felt they lacked sufficient knowledge on vaccine composition and needed more resources providing information adequately answering parent questions on this topic. This finding, however, is consistent with the literature that indicates parents who tend to exempt their children from school entry vaccines are often highly educated (Wang et al., 2014) and are prone to ask for more detailed information.

Participants concurred that creating a positive atmosphere that encouraged open dialogue between parents and health educators was another crucial element of best practice. These findings are consistent with those of Navin et al. (2018) who found that vaccine educators view a respectful, information-rich exchange—and not vaccine compliance—as the primary objective of the sessions. It is noteworthy to mention that the focus away from vaccine compliance may also be due in part to the inability to measure their impact on parents who do decide to vaccinate their child. This may be attributed to several reasons, one being that many health departments could not issue vaccines immediately after the education session, as they were not equipped to administer vaccines on site. Others could not bill for vaccination through private insurance. At the time of this study, there was no data collected at these sites indicating whether a child received a vaccine elsewhere following the vaccine education session. Nevertheless, the approach of cultivating a positive atmosphere remained central to each educator's intervention strategy.

Some participants suggested that the added responsibility of facilitating educational sessions could affect educators’ workload at some health departments; the strategy deemed most effective in addressing this issue was making the time required for obtaining a vaccine exemption the same as for receiving an immunization. Although this strategy may be effective in some districts, it is unclear from the interviews how well it would transfer to those areas experiencing both high vaccine exemption rates and limited personnel; indeed, some participants spoke of this as a barrier to the effective implementation of the sessions.

This study's findings contrast with those reported by Navin et al. (2018), in which educators reported feeling unprepared to communicate effectively with parents. A majority of participants in this study expressed feeling adequately trained and prepared for the session, despite a few citing a lack of confidence in answering parents’ questions regarding vaccine ingredients. The overall feeling of being adequately trained as expressed by participants in this study suggests that vaccine waiver educators may be quickly adapting to the role of servicing vaccine-hesitant parents and have identified which strategies are helping to ease some of the anxiety that arises while meeting with parents who are not at the health department by choice (Paterson et al., 2016).

## External Challenges to Implementing the Vaccine Waiver Intervention

State recommendations discouraging educators from dialoguing with parents regarding religious exemptions may unintentionally undermine the objectives of the program, in that, as stated by participants in this study, parents with philosophical objections might instead choose to ask for a religious exemption knowing that it is the path of least resistance to avoid discussion with a health official. This is a finding echoed in the 2018 study by Navin et al.

One notable challenge outlined in this study is the role of schools and their ability to be noncompliant with public health efforts. This study highlighted the need for schools and health department officials to work harmoniously when striving to uphold public health efforts. Simply engaging schools as stakeholders in the program planning and overall process may be a means to overcome this challenge, thus supporting the program's goals (Lillvis et al., 2020).

## Conclusion

Several states have instituted education interventions aimed at reducing the number of nonmedical exemptions; still, researchers, including Sadaf et al. (2013), suggest that there remains a persistent gap in the literature addressing effective interventions that target vaccine hesitancy. Charging local health departments with presiding over vaccine waiver education for vaccine-hesitant parents via a face-to-face discussion is a novel intervention strategy employed in Michigan. The implementation of this unique vaccine educational effort by participants in this study may not only have statewide application implications but can feasibly translate into national and international contexts. Furthermore, the strategies and techniques outlined by participants in this study have the potential to inform current COVID-19 vaccine initiatives, especially those targeting parents who are vaccine-hesitant about COVID-19 vaccines for their children.

Tasking local health departments with taking the lead in the vaccine exemption process seems unlikely to change in Michigan, as it has largely accomplished its intended purpose, which was to guarantee that parents seeking vaccine waivers for their children received education on the risks of not receiving the vaccines being waived and the benefits of vaccination to the individual child and the community. Therefore, sharing with vaccine educators those resources, strategies, and techniques that help promote positive and meaningful interaction during face-to-face encounters with parents is vital to addressing vaccine hesitancy.

### Strengths & Limitations

The main strength of this study lies in its use of qualitative methodology to enhance understanding of how the educational intervention targeting vaccine-hesitant parents at the local health departments is being conducted. Additionally, this study was able to furnish more than a general sense of which strategies educators currently utilize to reach vaccine-hesitant parents; it also allowed participants to detail which techniques they used to certify these strategies were well employed. This study also provides insight into how vaccine educators evaluate the training they received for undertaking this role of speaking to vaccine-hesitant parents.

While educators reported having had access to a wealth of resources, this study revealed which resources participants used, how they used them, and why; such inquiries were central to this study. Another notable strength of this study was that it conducted individual interviews with educators, which provided participants a platform of their own to freely discuss experiences and state opinions without any direct influence from their peers, thus minimizing bias.

The study was conducted while participants were at work, and this may be viewed as a limitation, as participants may not have felt they were at liberty to criticize the process over the telephone from their place of employment. Another noteworthy limitation of the study was its inability to assess the efficacy of the face-to-face, health department-based vaccine education intervention in improving vaccination rates among the children of parents who received education from participants. To the authors’ knowledge, at this time, local health departments do not collect this data. This is a remaining gap in the literature, but also points to the difficulty educators have in evaluating their interventions. Due to the voluntary nature of the study, the probability of selection bias is another limitation in that the sample most likely consisted of those who were most supportive of the process. Moreover, diversity among participants was lacking in that only four counties participated, meaning that some of the participants were from the same health department. However, this study illuminates which strategies and resources the educators who participated in this study have found most beneficial when speaking to vaccine-hesitant parents.

### Recommendations

In the interest of supporting vaccine waiver educators, state-level health officials may want to continue emphasizing open communication strategies and provide a diverse pool of resources from which educators may draw. The study pointed to the importance of providing vaccine educators with ongoing specific training and information that prepares them to respond to parents who voice concerns over specific vaccine ingredients. The authors also recommend the urgent study of how the recommendation from the state prompting vaccine educators to no longer challenge religious vaccine exemptions has affected religious vaccine exemption rates. Another recommendation is that the state ensures that all schools statewide are adhering to the public health code concerning first-day exclusion for individuals without proper vaccine documentation. Lastly, to address an incidental finding, we recommended that the state seek to determine a means by which educators might measure their impact on parental decision-making or receive feedback regarding their intervention efforts.
